# Supplementary figures and images for: Natural variation in the roles of C. elegans autophagy components during microsporidia infection
Source: PLoS One. 2019 Apr 23;14(4):e0216011. doi: 10.1371/journal.pone.0216011 (PMC6478341; doi:10.1371/journal.pone.0216011)

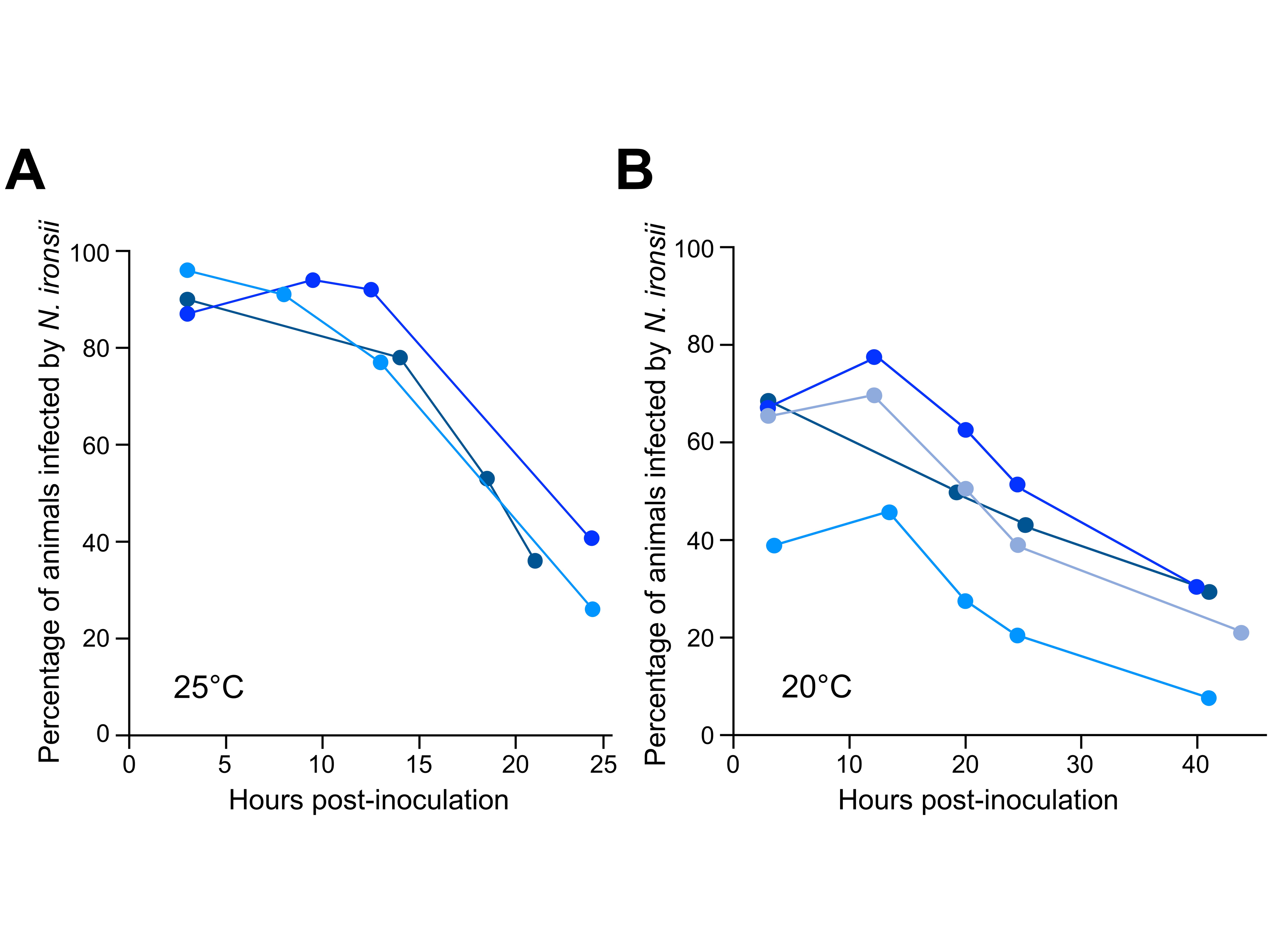

Supplement: S1 Fig — (A) HW L1 animals were pulse-inoculated with N. ironsii spores and fractions of the population were fixed at several time points afterwards to assess the frequency of infection. Experiments were carried out at 25°C. Data from three independent experiments are shown in blue lines. (B) Same experimental setup described above in (A), only the four experiments were carried out at 20°C. (TIF) [file pone.0216011.s001.tif]

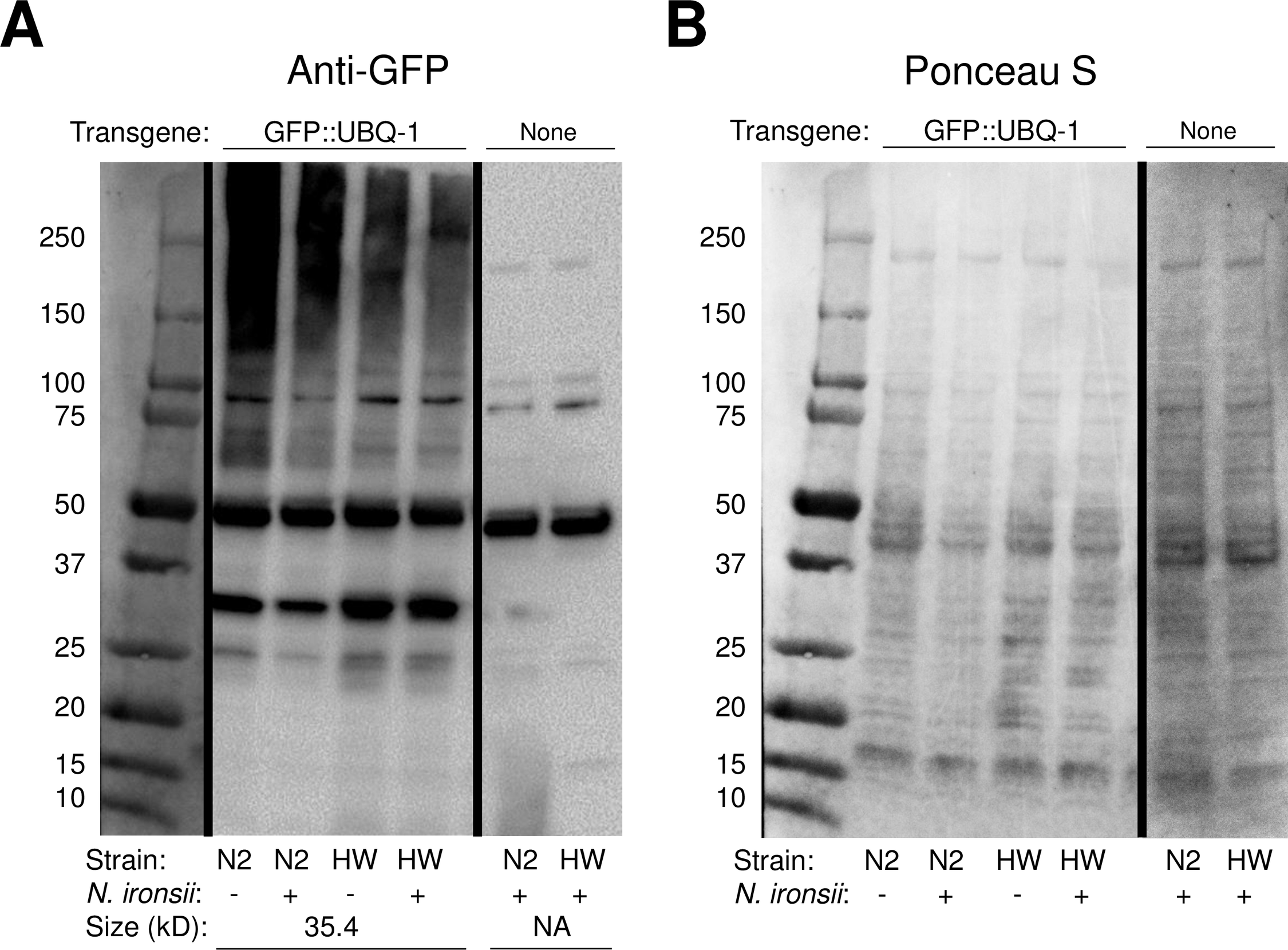

Supplement: S2 Fig — (A) Proteins were extracted from uninfected and N. ironsii infected animals (15 hpi) and analyzed by SDS-PAGE followed by Western blot using an anti-GFP antibody. Background staining of proteins from non-transgenic infected animals is shown in the last two columns. The predicted size of GFP::UBQ-1 is 35.4 kD. (B) Loading of all proteins per sample visualized by Ponceau S staining. (TIF) [file pone.0216011.s002.tif]

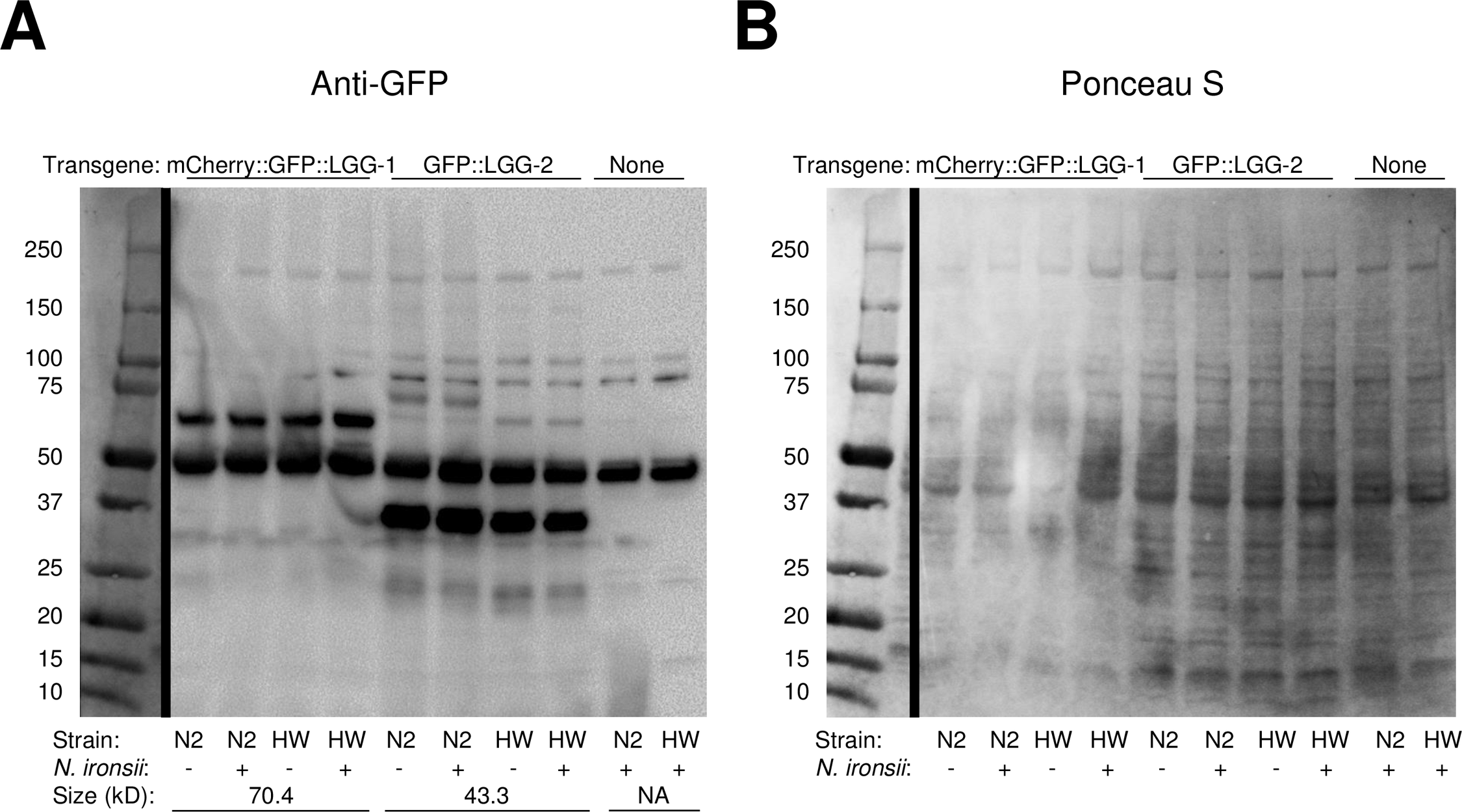

Supplement: S3 Fig — (A) Proteins were extracted from uninfected and N. ironsii infected animals (15 hpi) and analyzed by SDS-PAGE followed by Western blot using an anti-GFP antibody. Background staining of proteins from non-transgenic infected animals is shown in the last two columns. The predicted size of GFP::LGG-1 is 70.4 kD and the predicted size of GFP::LGG-2 is 43.3 kD. (B) Loading of all proteins per sample visualized by Ponceau S staining. (TIF) [file pone.0216011.s003.tif]

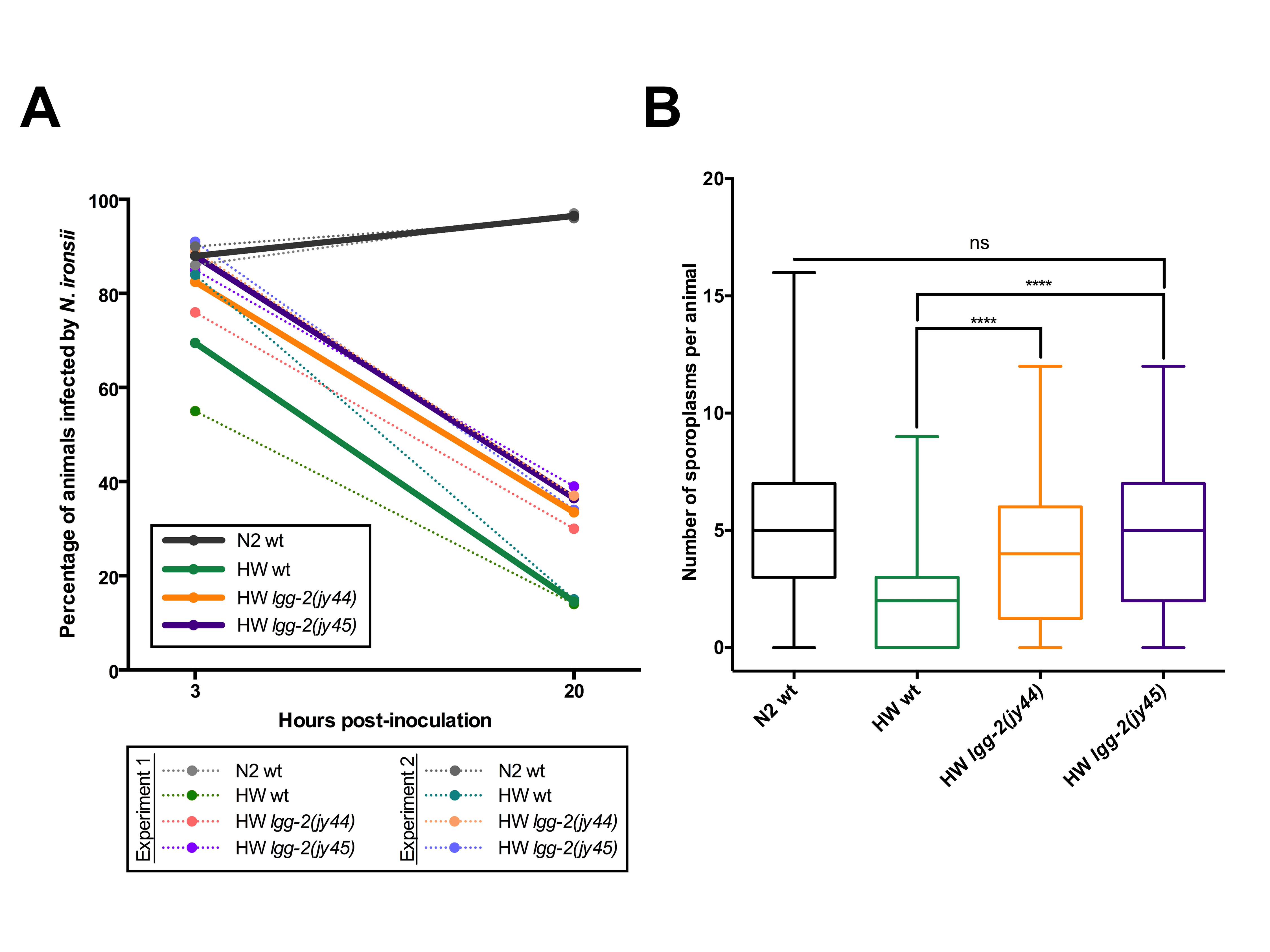

Supplement: S4 Fig — (A) N. ironsii clearance in lgg-2(jy44) mutants (orange line) and lgg-2(jy45) mutants (purple line). Thick lines represent average values from two independent experiments; each replicate is shown with a dotted line. HW and N2 wild-type controls are shown in green and gray lines, respectively. (B) Box-and-whiskers plot shows similar N. ironsii infection rate between lgg-2(jy44) and lgg-2(jy45) alleles (3 hpi). Each box represents 50% of the data from two independent experiments closest to the median value (line in the box). Whiskers span the values outside of the box. A student’s t-test was used to calculate p values; p < 0.001 is indicated with four asterisks; ns indicates non-significant difference (p > 0.05). (A, B) All experiments were performed at 25°C. (TIF) [file pone.0216011.s004.tif]

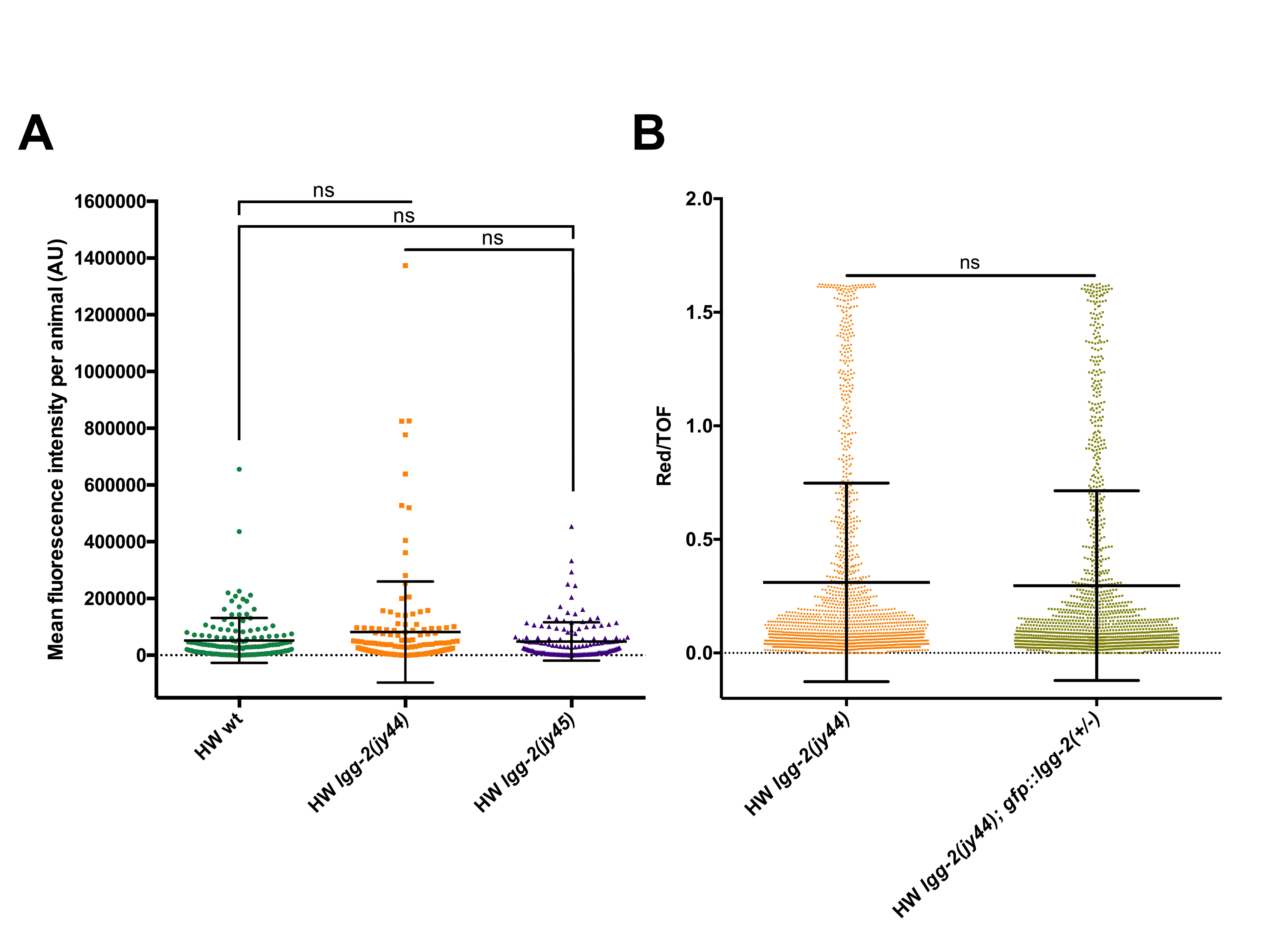

Supplement: S5 Fig — (A, B) Fluorescent bead accumulation in the intestines of infected L1 animals after 30 minutes. (A) lgg2(jy44) and lgg-2(jy45) mutants show similar feeding rate to HW wild-type animals. Feeding assay was performed in triplicate, 50 animals were analyzed per strain per experiment. Average red fluorescence intensities per whole animal are shown in arbitrary units (AU) on y-axis. Each dot represents one animal. (B) Mixed population of GFP::LGG-2 rescued and non-rescued lgg-2(jy44) animals have a similar feeding rate as lgg-2(jy44) strain. Fluorescence levels measurements were standardized to the body length for each animal. More than 1350 animals were analyzed from three individual experiments combined. (A, B) A student’s t-test was used to calculate p values; ns indicates non-significant difference (p > 0.05). All experiments were performed at 25°C. (TIF) [file pone.0216011.s005.tif]

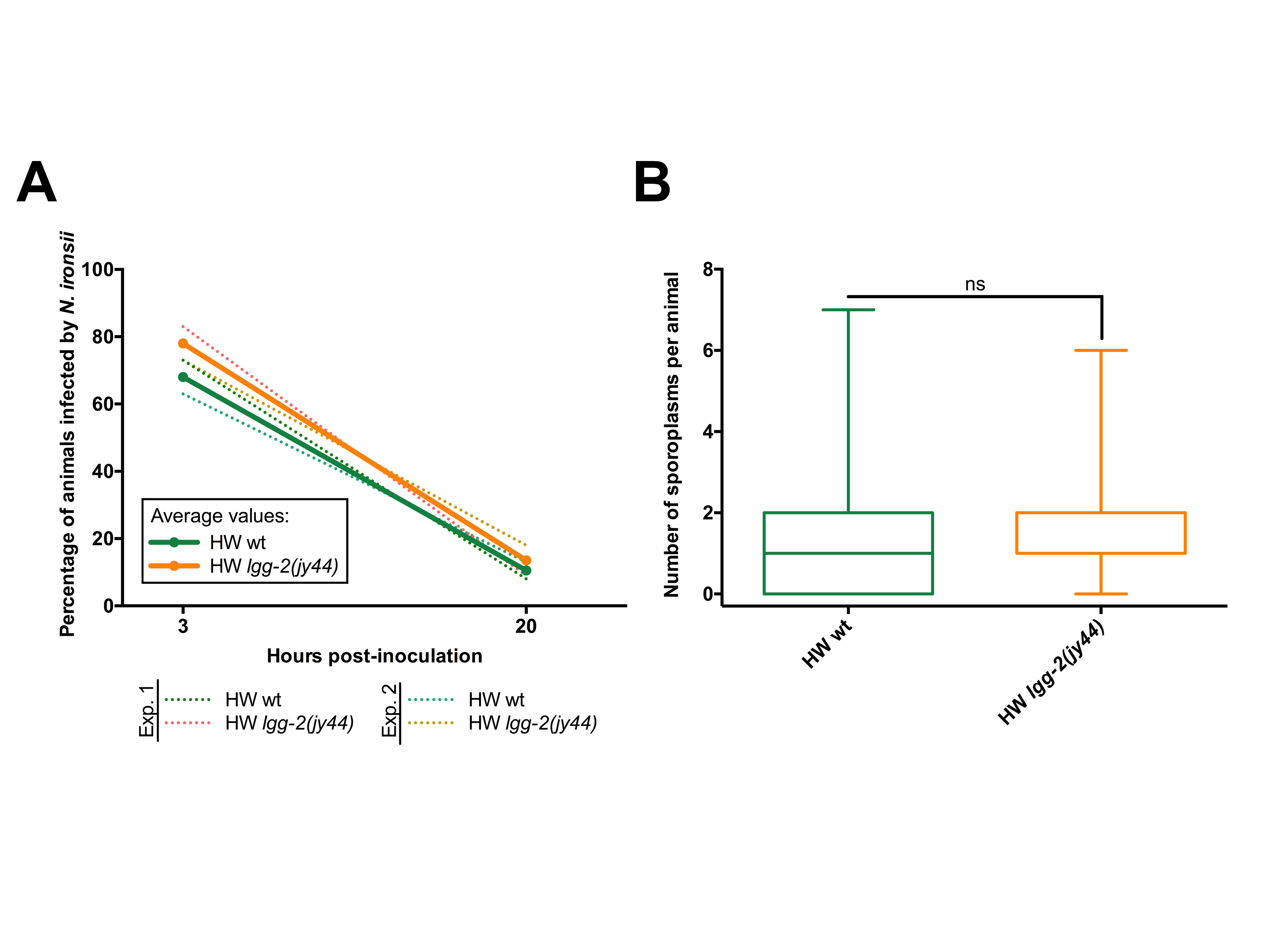

Supplement: S6 Fig — (A, B) N. ironsii clearance in HW wild-type and lgg-2(jy44) animals is similar if HW wt animals are infected with a higher dose of microsporidia spores than HW lgg- 2(jy44) mutants, to achieve similar initial intestinal colonization in both strains. (A) N. ironsii clearance in HW wild-type animals (green lines) and lgg-2(jy44) mutants (orange lines). Thick lines represent average values of two experiments; dotted lines indicate results from individual experiments. 100 animals were analyzed per strain at 3 hpi and 20 hpi (x-axis). (B) Box-and-whiskers plot shows similar N. ironsii infection rate between HW wild-type and lgg-2(jy44) mutant animals. Each box represents 50% of the data closest to the median value (line in the box). Note that the median value for HW lgg-2(jy44) sample is two sporoplasms per animal and that it overlaps with the upper boundary of the box. Whiskers span the values outside of the box. A student’s t-test was used to calculate p values; ns indicates non-significant difference (p > 0.05). (TIF) [file pone.0216011.s006.tif]

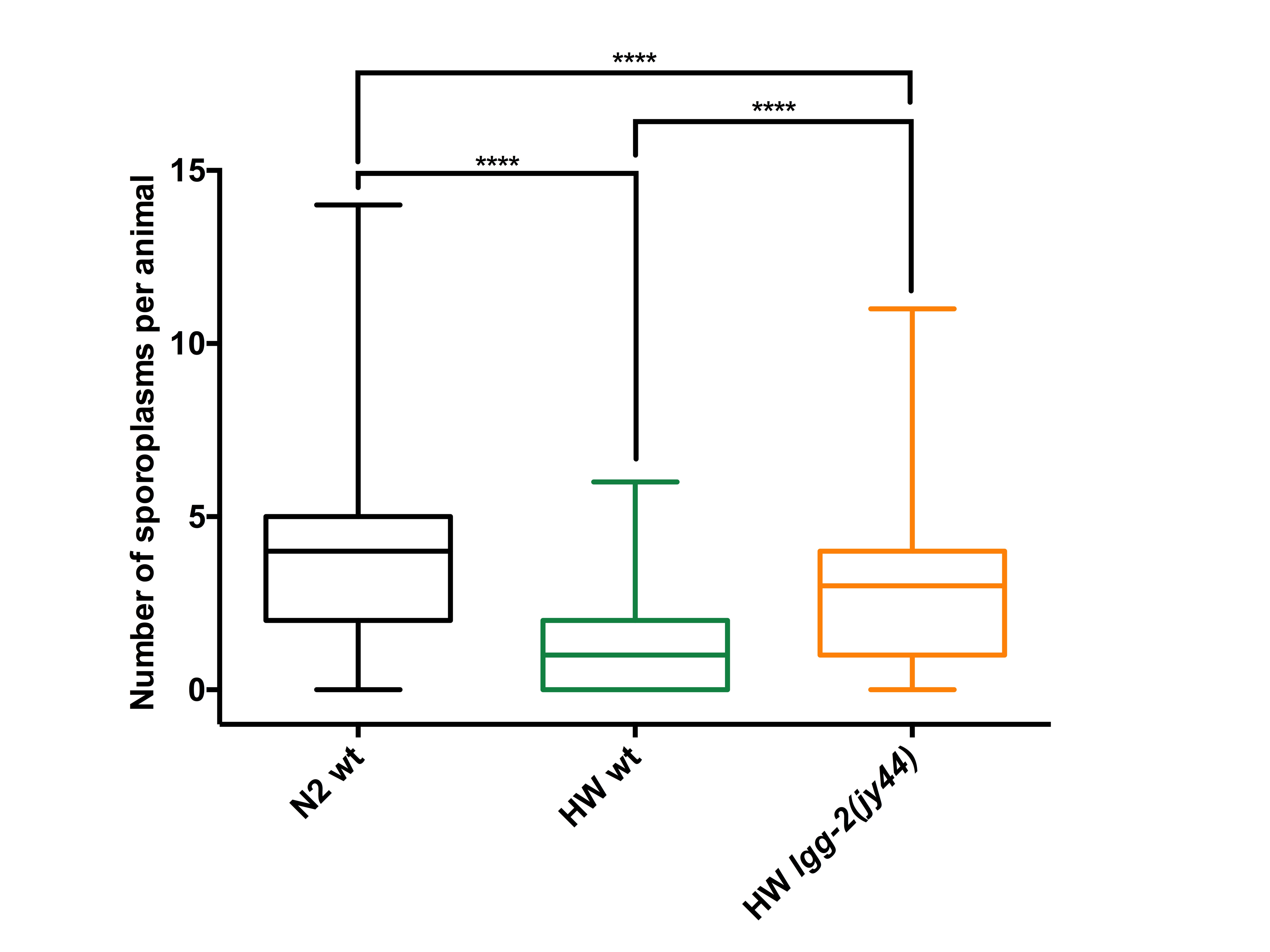

Supplement: S7 Fig — Significant difference in pathogen load is observed between HW wild-type and HW lgg-2 deletion mutant animals after 15 minutes incubation with 12 million N. ironsii spores (6 times more spores than in other clearance assays with lgg-2 mutants). Results from two independent experiments are shown as box-and-whisker plots, indicating the number of N. ironsii sporoplasms per animal at 3 hpi. Each box represents 50% of the data closest to the median value (line in the box). Whiskers span the values outside of the box. 400 animals were analyzed for each strain. A student’s t-test was used to calculate p values; p < 0.001 is indicated with four asterisks; ns indicates non-significant difference (p > 0.05). Experiments were performed in liquid culture at 25°C. (TIF) [file pone.0216011.s007.tif]

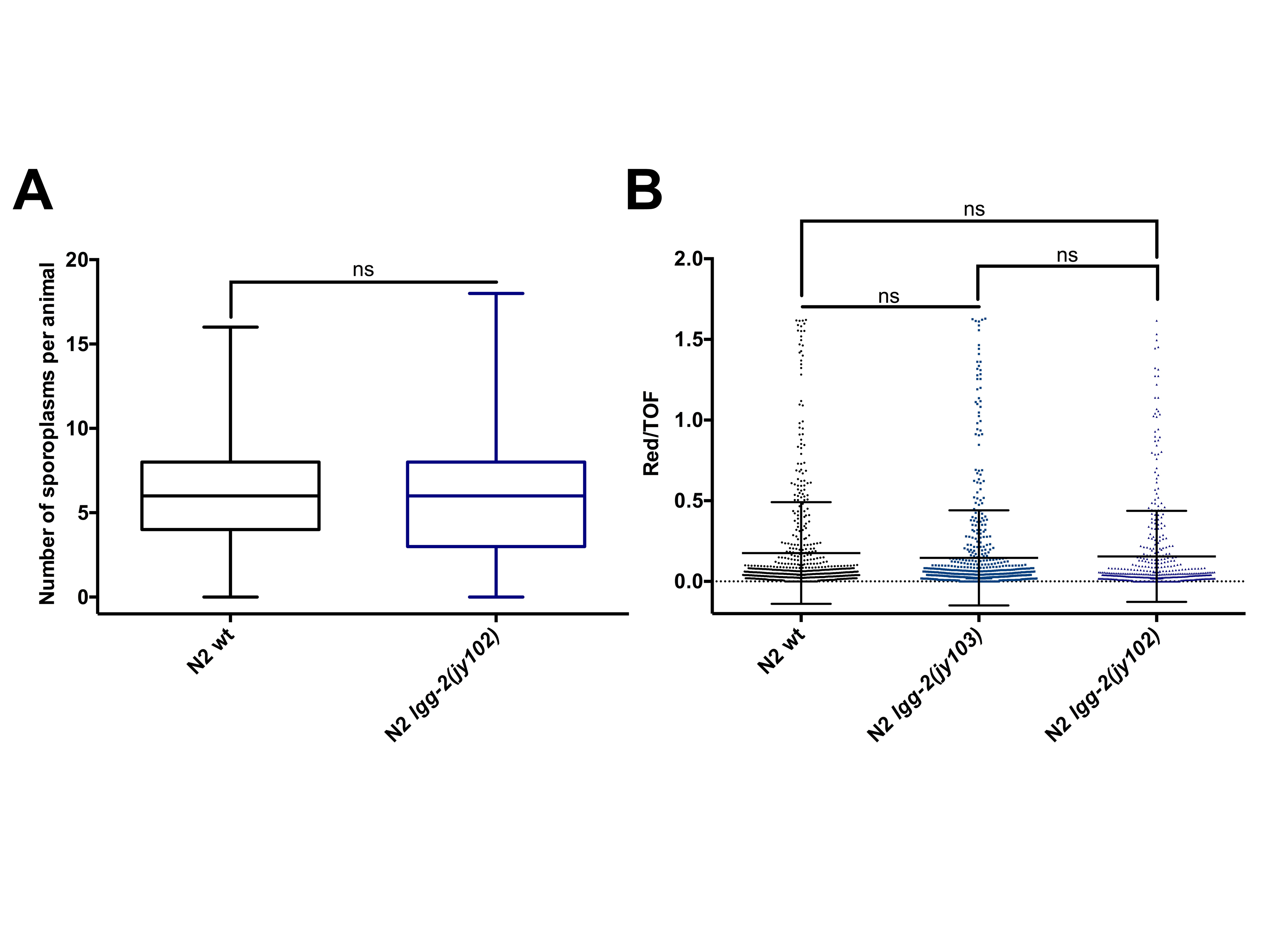

Supplement: S8 Fig — (A) lgg-2(jy102) mutants show similar infection rate as the wild type control. Results from two independent experiments are shown as box-and-whisker plots, indicating the number of N. ironsii sporoplasms per animal at 3 hpi. Each box represents 50% of the data closest to the median value (line in the box). Whiskers span the values outside of the box. 200 animals were analyzed for each strain. (B) N2 wild type and two N2 lgg-2 mutant strains (lgg-2(jy103) and lgg-2(102)) have similar feeding rate. Bead fluorescence is standardized to TOF. At least 450 animals were analyzed for each strain, combined from three replicate experiments. (A, B) A student’s t-test was used to calculate p values; ns indicates non-significant difference (p > 0.05). Experiments were performed at 25°C. (TIF) [file pone.0216011.s008.tif]
